# Glycolysis and beyond in glucose metabolism: exploring pulmonary fibrosis at the metabolic crossroads

**DOI:** 10.3389/fendo.2024.1379521

**Published:** 2024-05-24

**Authors:** Yuejiao Wang, Xue Wang, Chaoqi Du, Zeming Wang, Jiahui Wang, Nan Zhou, Baohua Wang, Ke Tan, Yumei Fan, Pengxiu Cao

**Affiliations:** ^1^ Ministry of Education Key Laboratory of Molecular and Cellular Biology, Hebei Key Laboratory of Animal Physiology, Biochemistry and Molecular Biology, College of Life Sciences, Hebei Normal University, Hebei Research Center of the Basic Discipline of Cell Biology, Hebei Collaborative Innovation Center for Eco-Environment, Shijiazhuang, Hebei, China; ^2^ Department of Laboratory, Hebei Provincial People’s Hospital, Shijiazhuang, Hebei, China; ^3^ Department of Gynecology, Xingtai People’s Hospital, Xingtai, Hebei, China; ^4^ Department of Thoracic Surgery, The Second Hospital of Hebei Medical University, Shijiazhuang, Hebei, China

**Keywords:** pulmonary fibrosis, glucose metabolism, glycolysis, TGF-β, diabetes mellitus

## Abstract

At present, pulmonary fibrosis (PF) is a prevalent and irreversible lung disease with limited treatment options, and idiopathic pulmonary fibrosis (IPF) is one of its most common forms. Recent research has highlighted PF as a metabolic-related disease, including dysregulated iron, mitochondria, lipid, and glucose homeostasis. Systematic reports on the regulatory roles of glucose metabolism in PF are rare. This study explores the intricate relationships and signaling pathways between glucose metabolic processes and PF, delving into how key factors involved in glucose metabolism regulate PF progression, and the interplay between them. Specifically, we examined various enzymes, such as hexokinase (HK), 6-phosphofructo-2-kinase/fructose-2,6-bisphosphatase 3 (PFKFB3), pyruvate kinase (PK), and lactate dehydrogenase (LDH), illustrating their regulatory roles in PF. It highlights the significance of lactate, alongside the role of pyruvate dehydrogenase kinase (PDK) and glucose transporters (GLUTs) in modulating pulmonary fibrosis and glucose metabolism. Additionally, critical regulatory factors such as transforming growth factor-beta (TGF-β), interleukin-1 beta (IL-1β), and hypoxia-inducible factor 1 subunit alpha (HIF-1α) were discussed, demonstrating their impact on both PF and glucose metabolic pathways. It underscores the pivotal role of AMP-activated protein kinase (AMPK) in this interplay, drawing connections between diabetes mellitus, insulin, insulin-like growth factors, and peroxisome proliferator-activated receptor gamma (PPARγ) with PF. This study emphasizes the role of key enzymes, regulators, and glucose transporters in fibrogenesis, suggesting the potential of targeting glucose metabolism for the clinical diagnosis and treatment of PF, and proposing new promising avenues for future research and therapeutic development.

## Introduction

Pulmonary fibrosis (PF) is a prevalent and irreversible pathology of lung tissue commonly observed in clinical practice. A variety of known factors, including exposure to toxic substances, autoimmune diseases, pharmacological agents, infectious injuries, or physical trauma, can contribute to PF (1). PF may also arise from unidentified causes, which is called idiopathic pulmonary fibrosis (IPF) and is one of the most common forms of PF (2). The pathological progression of PF is marked by the accumulation of excessive extracellular matrix (ECM), resulting from the excessive proliferation and fibrotic differentiation of activated fibroblasts, accompanied by inflammatory responses and varying degrees of structural damage. Oxidative stress and epithelial-mesenchymal transition (EMT) also promote the pathological progression of PF. PF leads to a severe decline and continuous deterioration of respiratory function, characterized by symptoms such as a persistent dry cough and progressive dyspnea. Existing anti-fibrotic medications only delay the progression of mild to moderate PF and are associated with obvious side effects. Due to its unclear etiology, extremely poor prognosis, and lack of truly effective drugs, PF become a hot but challenging research spot.

In recent years, PF has been increasingly recognized as a metabolism-related disease. Beyond lipid metabolism, an increasing number of studies have shown that IPF is associated with imbalanced glucose metabolism, and alterations in glucose metabolism play an important role in the development of PF (3–5). Some molecules participating in glucose metabolism also play roles in PF. Glucose metabolism encompasses aerobic oxidation and glycolysis. During aerobic oxidation, one molecule of glucose is completely oxidized to produce 36 (or 38) molecules of ATP, the primary energy source in the body. Under anaerobic conditions, glucose is converted to lactate through glycolysis, yielding 2 molecules of ATP. This report delves into how important factors mediate the interaction between glucose metabolism and PF, focusing on the key molecules involved in glycolysis, glucose metabolism or diabetes mellitus that play regulatory roles in lung fibrosis. The aim is to unravel the intrinsic associations between glucose metabolism and lung fibrosis, providing novel insights for the development of truly effective therapeutic strategies for PF.

## Key enzymes and metabolite in glycolysis play regulatory roles in pulmonary fibrosis

### Hexokinase (HK)

HK, the primary rate-limiting enzyme in glycolysis, consists of four isozymes: HK1–4. HK2 is insulin-sensitive and is predominantly found in adipose and muscle tissues. Yin et al. reported that increased HK2 expression has been observed in fibroblasts of patients with IPF and in bleomycin-induced fibrotic lungs in mice (3). HK2 plays a pivotal role in mediating the fibroproliferative activity of transforming growth factor-beta (TGF-β) and YAP/TAZ, and the induction of HK2 is mediated by Smad-dependent and Smad-independent pathways. Both *HK2 siRNA* and a mitochondrial HK inhibitor, lonidamine, reduced TGF-β-induced expression of fibrogenesis-related proteins, including collagen, fibronectin, and smooth muscle actin alpha 2 (α-SMA) in human lung fibroblasts and mouse fibroblasts. *In vivo*, administration of lonidamine to bleomycin-induced mouse reversed upregulation of pulmonary Hk2, reduced molecular markers of PF, and enhanced lung function (3). In addition, the glucose analog 2-deoxy-D-glucose, an inhibitor of HK2, inhibits glycolysis and collagen synthesis in lipopolysaccharide (LPS)-induced human lung fibroblasts (5, 6).

### 6-Phosphofructokinase-2-kinase/fructose-2,6-bisphosphatase 3 (PFKFB3)

PFKFB3 catalyzes the conversion of fructose-6-phosphate into fructose-2,6-bisphosphate, and the latter is an allosteric activator of phosphofructokinase 1 (PFK1), the second rate-limiting enzyme in glycolysis, thus significantly enhancing glycolysis in the cells (7, 8). Previous research has highlighted that the elevation in aerobic glycolysis is a crucial step in the activation of myofibroblasts (9). In bleomycin-induced pulmonary fibrosis model, anlotinib exerts anti-fibrotic effects by inhibiting glycolysis in myofibroblasts mediated by downregulation of RNA-binding protein, poly (rC) binding protein 3 (PCBP3), and the subsequent translation reduction of PFKFB3 (10). In the experimental PF mouse model and human pulmonary fibroblast MRC-5 cell line, LPS significantly upregulated PFKFB3 expression by activating the PI3K/Akt/mTOR pathway, thereby augmenting glycolysis and promoting collagen synthesis. These effects of LPS can be reversed by the addition of PFKFB3 inhibitor, 3PO (5). Consequently, PFKFB3 emerges as a pivotal regulator of glycolysis and an essential therapeutic target in IPF.

### Pyruvate kinase (PK)

PK, the final rate-limiting enzyme in the glycolysis pathway, catalyzes the conversion of phosphoenolpyruvate into pyruvate and ATP. PK comprises four isoenzymes: PKL, PKR, PKM1, and PKM2. PKM1 is primarily expressed in muscle and brain tissues, whereas PKM2 is predominantly found in stem cells, embryonic cells, and tumor cells. In silica-induced alveolar macrophages and silicosis models, elevated levels of PKM2 were observed (11). Notably, PKM2 is recognized as a critical regulator of glycolysis in tumor cells, playing a vital role in regulating the transcription of glycolysis-related genes, including *lactate dehydrogenase A (LDHA)*, *pyruvate dehydrogenase kinase (PDK* ***)***, and *glucose transporter 1 (GLUT1)* (12).

### Lactate dehydrogenase (LDH)

LDH catalyzes the final step of glycolysis, converting pyruvate into lactic acid. The isoenzymes of LDH are encoded by three genes: *LDHA, LDHB*, and *LDHC*, and are present in the cytoplasm of almost all tissues. High levels of lactate and LDH expression in the lung tissues of patients with IPF (13) suggest a shift in glucose metabolism toward lactate production. Elevated levels of LDHA and lactate have been observed in silica-induced silicosis models in mice and rats (11, 14). Additionally, the silica-induced enhancement of glycolysis and macrophage activation was diminished by *Ldha siRNA* treatment, highlighting that a metabolic shift to glycolysis is a crucial factor in the progression of silicosis fibrosis (11). Administration of an effective LDHA inhibitor, oxamate, in silicosis-afflicted mice reduced lactate levels, and exhibited anti-fibrotic effects via glycolysis and endoplasmic reticulum stress modulation (14). These findings suggest that LDHA could be a promising therapeutic target for the treatment of IPF.

### Lactate

Normally differentiated cells primarily utilize mitochondrial oxidative phosphorylation for their cellular energy supply, whereas most tumor cells rely on anaerobic glycolysis, converting glucose into lactate, a process known as the “Warburg effect.” The study by Cui et al. demonstrated that lactate in the extracellular environment of myofibroblasts stimulates macrophages, thereby promoting fibrogenesis (15). Research by Kottmann et al. confirmed that lactate induces myofibroblast differentiation via pH-dependent activation of TGF-β (16). Notably, while extracellular activation of TGF-β generally occurs under extreme pH conditions, physiological concentrations of lactate activate TGF-β in a dose-dependent manner. TGF-β stimulates the expression of lactate dehydrogenase-5 (LDH5) through hypoxia-inducible factor 1 subunit alpha (HIF-1α), thereby promoting the glycolytic process and subsequent production of additional lactate, which in turn enhances activation of TGF-β, resulting in upregulation of fibrotic markers such as α-SMA, and further progression of PF; conversely, simultaneously inhibiting both HIF-1α and LDH5 effectively suppresses TGF-β-induced myofibroblast differentiation (16).

### Pyruvate dehydrogenase kinase

PDK is critical for maintaining glucose homeostasis and includes four isoenzymes: PDK1–4. PDK4 is particularly notable due to its expression being significantly influenced by various physiological conditions, and inhibits the activity of the pyruvate dehydrogenase complex (PDC), thus redirecting pyruvate into the lactate metabolic pathway and facilitating the shift from oxidative phosphorylation to glycolysis (17). Goodwin et al. reported that elevated expression of PDK1 has been identified in patients with interstitial lung disease, according to microarray data analyzed from the Lung Genomics Research Consortium database (18). The overexpression of PDK1 activates glycolysis and enhances myofibroblast differentiation, marked by an accumulation of lactate, increased expression of α-SMA, and phosphorylation of pyruvate dehydrogenase (PDH). *PDK1 siRNA* in TGF-β-mediated myofibroblasts or the treatment of human lung fibroblasts with PDK1 inhibitor dichloroacetate, results in the dephosphorylation of PDH, reduced lactate levels, and a decrease in myofibroblast markers α-SMA and calponin 1. Notably, dichloroacetate markedly alleviates bleomycin-induced lung fibrosis. These findings highlight the role of PDK1-mediated glycolytic reprogramming as a critical metabolic change that promotes myofibroblast differentiation and fibrogenesis (18). The prevalence of PF is widely acknowledged to be closely related to aging. Studies have demonstrated an increase in PDK4-dependent aerobic glycolysis and lactate production in senescent cells. Lactate stimulates the generation of reactive oxygen species through NADPH oxidase 1 (NOX1), thus contributing to the progression of the senescence-associated secretory phenotype. In preclinical trials, the inhibition of PDK4 has been shown to alleviate physical dysfunction and prevent age-related frailty (19).

### Glucose transporters (GLUTs)

GLUTs constitute the most extensive family of membrane transport proteins, primarily responsible for transporting glucose into cells in a concentration gradient-dependent manner without consuming energy. Currently, 14 types of GLUTs have been identified, encompassing GLUT1–12, the H^+^- dependent myo-inositol transporter (HMIT), and GLUT14. GLUTs regulate glucose metabolism, inflammatory responses, and immune reactions, exhibiting high sensitivity and regulated by signals from the intracellular environment, small molecules, and metabolites. Both carcinogenic transformation and hypoxia induce the transcription of *Glut1* (20). Elevated levels of GLUT1 expression have been observed in patients with IPF as well as in bleomycin-induced mouse models of lung fibrosis. TGF-β induces GLUT1 expression in lung fibroblasts, a process mediated via the classical Smad2/3 pathway and involving the activation of PI3K, MEK, and mammalian target of rapamycin complex 2. Inhibition of GLUT1 activity and/or expression disrupts the TGF-β-driven fibrogenic process, including cell proliferation and the production of profibrotic mediators (21). Notably, GLUT1-dependent glycolysis is more active in older mice compared to their younger counterparts. Moreover, adenosine 5’-monophosphate (AMP)-activated protein kinase (AMPK), representing a critical metabolic pathway for fibroblast activation, is regulated by GLUT1-dependent glycolysis. Genetic and pharmacological suppression of Glut1 has been shown to down-regulate α-SMA in primary mouse lung fibroblasts. Phloretin, an effective inhibitor of GLUT1, significantly mitigates bleomycin-induced lung fibrosis *in vivo*. Consequently, GLUT1 inhibition presents a potential therapeutic avenue for the treatment of PF (22).

## Critical regulatory factors mediate the cross-talk between pulmonary fibrosis and glucose metabolism

### TGF-β

Some key regulatory factors in IPF also play critical roles in glucose metabolism. TGF-β affects the balance and function of multiple endocrine signaling pathways, including adrenocortical steroidogenesis (23), insulin signaling (24), the thyroid hormone pathway (25), and sex hormone signaling (26), etc. TGF-β, an essential cytokine that induces fibrosis in cells and tissues, is induced in human fibrotic lungs, as well as in experimental animal models of lung fibrosis (27). In models of lung fibrosis induced by bleomycin, M2-type macrophages produce fibrogenic cytokines, including TGF-β1, which trigger the activation of fibroblasts and promote the differentiation of myofibroblasts (28). TGF-β family consists of structurally and functionally related subfamilies of peptide growth factors, including three isoforms: TGF-β1, TGF-β2, and TGF-β3. The TGF-β pathway, a central signaling pathway in the development of IPF, is involved in regulating inflammatory responses, fibroblast proliferation, differentiation, invasion, excessive ECM secretion, EMT, and characterizes a typical membrane-to-nucleus signal transduction process. TGF-β binds to its type II receptor, thereby triggering the phosphorylation of its type I receptor. The receptor-activated Smad (R-Smad) dissociates from the type I receptor and forms a complex with Smad4. This complex is then transported to the nucleus, where it interacts with transcription factors to regulate the expression of TGF-β target genes (29). In addition, TGF-β can also activates the MAPK signaling pathway and PI3K/Akt signaling pathway through non-Smad signal pathways, and Wnt/β-catenin signaling, further regulating IPF (30–32).

TGF-β1 upregulates glycolysis in many organs, such as the liver and kidney (25, 33). During tumorigenesis, TGF-β induces a shift in cell metabolism from oxidative phosphorylation to aerobic glycolysis (34). In addition to the positive feedback relationship between TGF-β and lactate, TGF-β1 enhances the expression of PFKFB3, HK2, GLUT1, and LDHA, leading to increased glucose uptake, glycolysis, extracellular acidification rate, and oxygen consumption rate in fibroblasts (35, 36). In the absence of glucose or in the presence of the glycolytic inhibitor 2-deoxyglucose, TGF-β1-induced collagen synthesis is significantly reduced, underscoring the essential role of glycolysis in TGF-β1-induced collagen deposition (37). The study by Wu et al. has shown that when fibroblasts and epithelial cells are treated with high glucose, the cell surface content of transforming growth factor beta receptor 1 (TβRI) and transforming growth factor beta receptor 2 (TβRII) increases, activating TGF-β through matrix metalloproteinases (including MMP2 and MMP9), thereby activating the Akt/mTOR pathway and leading to fibroblast hypertrophy. Inhibiting MMP2/MMP9 or TGF-β-induced mTOR activation can suppress high glucose-induced cell hypertrophy. The presence of neutralizing anti-TGF-β antibody reduces the increase in collagen synthesis in glucose-induced glomerular mesangial cells (38). TGF-β induces metabolic reprogramming of lipid metabolism during the EMT process, increasing glycolysis and fatty acid synthesis (39). Studies also reveal that in patients with Type 2 diabetes and under high glucose treatment *in vitro*, the expression of TβRII in monocytes is upregulated, enhancing TGF-β pathway signaling and inhibiting vascular endothelial growth factor receptor-mediated monocyte migration, thus leading to vascular dysfunction in Type 2 diabetes (40).

### Interleukin-1 beta (IL-1β)

IL-1β influences the secretion of various hormones, including adrenocorticotropic hormone, cortisol, growth hormone and insulin, and is involved in a wide range of physiological and pathological processes (41–43). IL-1β is a proinflammatory and profibrotic cytokine and primarily produced by mononuclear macrophages. IPF macrophages produce excessive IL-1β upon the stimulation of NLRP3, AIM2 and NLRC4 inflammasomes; IL-1β initiates and amplifies lung inflammation and has been associated with acute lung injury and fibrosis (44). An increase in IL-1β levels has been observed in the bronchoalveolar lavage fluid and homogenized lung supernatant from bleomycin-induced fibrotic mice and patients with IPF; intratracheal administration of IL-1β induces significant pulmonary inflammation in mice, characterized by notable neutrophilia in the bronchoalveolar lavage, along with IFN-γ and IL-17A production in local lymph nodes, leading to pulmonary fibrosis. This response is highly dependent on IL-17A (45). Exogenous neuropeptide Y treatment has been found to reduce pulmonary IL-1β concentrations and inhibit the progression of bleomycin-induced pulmonary fibrosis (46).

Importantly, IL-1β promotes glycolysis via p38 pathway, which mediates growth and invasion in lung adenocarcinoma (47). IL-1β also mediates the increase of PFKFB3 by the NLRP3 inflammasome, which modulates glycolysis (48). Regarding glucose metabolism, mRNA levels of *Glut-2*, a principal hepatic glucose transporter, are reduced in the livers of mice with IL-1β-secreting tumors, thus promoting hypoglycemia (49). IL-1β plays a critical role in inflammation, which is central to Type 2 diabetes. IL-1β-mediated autocrine inflammation driven by glucose, free fatty acids, leptin, and IL-1β itself, leads to β-cell death; the balance between IL-1β agonist activity and the naturally occurring IL-1 receptor antagonist (IL-1Ra) determines the outcome of islet inflammation (50). High glucose levels can induce IL-1β production in β-cells from islets donated by diabetic patients, followed by NF-κB activation, Fas upregulation, DNA fragmentation, and β-cell dysfunction (51, 52).

### HIF-1α

HIF-1α is the primary regulator of cellular and systemic responses to hypoxia, functioning as a transcription factor. It activates the transcription of genes that mediate adaptive responses to hypoxia, including erythropoietin, vascular endothelial growth factor, and glycolytic enzymes (53), thus participating in critical processes of energy metabolism, angiogenesis, and cell apoptosis, and playing a central role in maintaining physiological balance in the body under hypoxic condition. HIF-1α enhances glycolysis in several types of cancers (54, 55). For instance, HIF-1α enhances glycolysis in esophageal cancer cells by upregulating HK2 and PDK1 expression under hypoxic conditions (56). By promoting glycolysis, HIF-1α assists cells in generating sufficient ATP to maintain survival, especially in tissues with inadequate blood supply. Additionally, HIF-1α can enhance glycolysis even in oxygen-sufficient conditions. Moreover, HIF-1α can also be induced by various stimuli, and is an essential mediator of endothelin in granulosa cells (57).

In lung tissues of IPF patients and in a bleomycin-induced mouse model of PF, HIF-1α expression is elevated (58). HIF-1α supports a pro-fibrotic phenotype loop with emphasis on SERPINE1, VEGFA and TIMP1 in IPF (59). By using myofibroblast-specific promoter-driven HIF-1α knockout mice, fibrosis is found to be reduced in a bleomycin-instilled PF model, indicating that HIF-1α plays a crucial role in promoting myofibroblast differentiation and the progression of fibrosis. Hypoxia significantly enhances TGF-β-induced myofibroblast differentiation, which is through HIF-1α/PDK1-mediated glycolytic reprogramming (18). Furthermore, HIF-1α up-regulates the ADORA2B receptor in alternatively activated macrophages and contributes to lung fibrosis, supporting a potential role for HIF-1α antagonists in the treatment of IPF (60).

### NADPH oxidase 4 (NOX4)

NOX4 is a subtype of the NOX family of NADPH oxidases, primarily expressed in the cytoplasm and endoplasmic reticulum of pulmonary vascular systems and lung endothelial cells (61). In lung fibroblasts of IPF patients, an increase in NOX4 expression at mRNA and protein levels has been detected and is positively correlated with the expression of *α-SMA* and *pro-collagen I (α1)* mRNA. NOX4-mediated ROS production is responsible for TGF-β-induced myofibroblast differentiation in lung fibroblasts (62). When *NOX4* expression is silenced by *siRNA* in lung fibroblasts from IPF patients and TGF-β-treated normal lung fibroblasts, the expression of α-SMA and pro-collagen I (α1) is inhibited, mediated by the activation of Smad2/3 transcription factors. NOX4 also mediates platelet-derived growth factor (PDGF)-induced fibroblast migration (63). NOX4 is also closely related to glycolysis; it directs glucose metabolism not only to glycolysis but also to the pentose phosphate pathway for NADPH production in NSCLC cells through ROS/PI3K/Akt-dependent c-Myc, and promotes glutamine breakdown into GSH for oxidative resistance, facilitating NSCLC growth; selective NOX4 inhibitor GKT137831 or elimination of NOX4-derived H_2_O_2_ effectively reverses the metabolic effects induced by NOX4 in NSCLC cells (64).

### Activating transcription factor 4 (ATF4)

ATF4 is a key protein in metabolic regulation, involved in the transcription of genes related to carbohydrate and lipid metabolism, energy homeostasis, amino acid biosynthesis and transport, antioxidant responses, and autophagy. ATF4 regulates insulin secretion and sensitivity. In mice deficient in *ATF4*, insulin levels are higher and the insulin content increases with glucose stimulation (65). ATF4 is highly expressed in myofibroblasts in the lungs of IPF patients (13). In lung myofibroblasts induced by TGF-β, ATF4 is activated via the PI3K/Akt/mTORC1 pathway (37, 66). The activation of ATF4 by TGF-β is essential for increasing the expression of serine-glycine synthesis pathway enzymes, including phosphoglycerate dehydrogenase (PHGDH), phosphoserine aminotransferase 1 (PSAT1), phosphoserine phosphatase (PSPH), and serine hydroxymethyltransferase 2 (SHMT2), as well as GLUT1 (37, 66, 67). Glycine, the most abundant amino acid in collagen, is synthesized *de novo* from serine, which is derived from the glycolytic intermediate 3-phosphoglycerate, and is essential for myofibroblasts to synthesize collagen (66). The inhibition of enzymes in the serine-glycine synthesis pathway inhibits the function of TGF-β-induced collagen production (67). Knocking out *ATF4* prevents the expression of serine-glycine synthesis pathway enzymes induced by TGF-β, thereby inhibiting collagen production in lung myofibroblasts (13). Tribbles pseudokinase 3 (TRIB3), a serine/threonine kinase-like protein, negatively regulates the ATF4 promoter-driven transcription activity and reduced ATF4 expression. Overexpression of TRIB3 inhibits fibroblast activation and reduces the synthesis and deposition of ECM in human embryonic lung fibroblasts, thereby mitigating PF (68).

### Advanced glycation end products (AGEs)

AGEs are stable compounds formed under non-enzymatic conditions through the condensation, rearrangement, cleavage, and oxidative modification of reducing sugars with free amino groups of proteins, amino acids, lipids, or nucleic acids. AGEs interact with their cell surface receptor, receptor for AGEs (RAGE), a member of the immunoglobulin superfamily of cell surface receptors. This interaction activates biological processes such as oxidative stress and inflammatory responses and is involved in complications related to diabetes, as well as some diseases including pulmonary fibrosis (69–71). Increased levels of AGEs have been detected in serum and lung tissues of IPF patients (70). RAGE is most highly expressed in the lungs compared to other tissues in healthy adult animals, but the ratio of membrane RAGE/soluble RAGE expression is decreased in lung tissues of IPF patients and in the asbestos mouse model for pulmonary fibrosis (72). The AGEs-RAGE axis induces upregulation of cytokines such as TGF-β1, TNF-α, and IL-8, playing a key role in NF-κB-dependent Type I collagen expression, and stimulating Type III collagen expression in tandem with TGF-β1 (71). Studies have confirmed that in naturally aging *RAGE-knockout* mice, pulmonary fibrosis is observed, and a worsening condition in cases of bleomycin-induced pulmonary fibrosis was demonstrated (72, 73). In a diabetic mouse model, global overexpression of phosphomimetic RAGE in the nucleus has been shown to restore DNA repair, thereby reducing DNA damage, inflammation, and pulmonary fibrosis (4).

## Diabetes mellitus and the closely related factors regulate pulmonary fibrosis

### Diabetes and hyperglycemia

The prevalence of diabetes among IPF patients is higher than that in the general population (74). Diabetes mellitus is one of the most common endocrine metabolic diseases, characterized by inflammation and oxidative stress. It is a chronic disease that is incurable but can be controlled. Diabetes is classified primarily into four types: Type 1 Diabetes, Type 2 Diabetes, Gestational Diabetes, and other types resulting from conditions such as pancreatitis or tumors in other parts of the body. As diabetes progresses or blood sugar levels remain elevated, there is consequent damage to blood vessels and other organs. Complications of diabetes include diseases affecting the eyes, kidneys, vascular system, and lungs (75). In patients with diabetes, there is significant thickening of the walls of alveolar capillaries, pulmonary arteries, and alveoli, with enhanced proliferation of extracellular connective tissue in the lungs and a significant decline in lung function (76, 77). For instance, reductions in lung volume parameters and diffusion capacity have been found in patients with type 2 diabetes (78). Meta-analyses have shown a positive correlation between diabetes and IPF, and revealed that type 2 diabetes is an independent risk factor associated with IPF (79). Similarly, elevated blood glucose levels are associated with an increased risk of IPF (80). Moreover, diabetes is considered a factor and marker for poor prognosis in IPF patients, and an increasing number of studies have found that certain anti-diabetic drugs have anti-fibrotic effects, such as the GLP-1 receptor agonist, liraglutide, which demonstrates anti-fibrotic effects in experimental animals (81).

Consistently, Ofulue et al. reported the occurrence of pulmonary fibrosis in streptozotocin (STZ)-induced diabetic rats, and the increase in lung collagen and elastin tended to normalize with insulin therapy (82). This evidence directly indicates that hyperglycemia leads to pulmonary fibrosis, expanding our understanding from merely an association between diabetes and IPF to a direct impact of diabetes on lung fibrosis. Pulmonary manifestations in STZ-induced diabetic mice include oxidative stress, apoptosis, edema, enhanced cell proliferation, honeycomb-like alveoli, thicker alveolar walls, microvascular leakage, and increased mRNA expression of fibrogenic markers such as *Acta2, Ccn2*, and *Fn1*, alongside pathological proliferation of extracellular matrix and interstitial connective tissue (83–87). Furthermore, the capillary basement membrane thickness is significantly increased in the alveolar septa of OVE26 transgenic diabetic mice (88). In mouse and rat models of both type 1 and type 2 diabetes, significant pulmonary fibrosis has been observed (89). *In vitro*, exposure of cultured L2 cells to high glucose levels resulted in increased CTGF expression associated with elevated STAT3 levels (79). Talakatta et al. reported upregulation of EMT and N-cadherin levels in diabetic cells exposed to high glucose concentrations (90). Due to the causal effects of diabetes on IPF, some researchers suggest substituting the term “idiopathic” pulmonary fibrosis in diabetic patients (when other causes of lung diseases are excluded) with the term “diabetes-induced pulmonary fibrosis” (DiPF) (89).

IPF and diabetes share similar pathological mechanisms, including oxidative stress, inflammation, endoplasmic reticulum stress, mitochondrial dysfunction, aging, and receptor-mediated signaling of glycosylated proteins (91). Mechanistically, diabetes induces cellular senescence which is accompanied by characteristic features of the senescence-associated secretory phenotype, including the release of pro-inflammatory cytokines and growth factors (89); high glucose levels induce local biochemical changes in the lungs, reducing lung antioxidant capacity; chronic hyperglycemia leads to glycosylation of immunoglobulins, increasing susceptibility to acute and chronic pulmonary infections; it can also lead to non-enzymatic glycosylation of collagen in the chest wall and bronchus, leading to fibrotic tissue formation (92). Persistent hyperglycemia can damage alveolar epithelial cells or increase the production of pro-inflammatory and pro-fibrotic factors (81), as well as induce lung EMT, promoting pulmonary fibrosis in experimental diabetes models (93).

Additionally, there are indirect interactions between hyperglycemia or diabetes and IPF through common risk factors, such as obesity, smoking, and cardiovascular diseases, or through a series of diabetes-related complications such as cardiovascular and kidney diseases, which may further increase the risk of developing IPF. For example, gastroesophageal reflux disease (GERD) is a known risk factor for IPF; the high prevalence of GERD in patients with either diabetes or IPF highlights a complex interplay between these conditions (75).

### AMPK

AMPK is found in various metabolically related organs and is activated when cells sense a lack of ATP, or when AMP level or AMP: ATP ratio rises. AMPK plays a pivotal role in regulating bioenergetics and is central to the study of diabetes and other metabolism-related diseases. Activating AMPK through drugs or exercise alleviates hyperglycemia and hyperlipidemia while enhancing insulin sensitivity (94). For example, metformin is an AMPK activator and a widely used medication for non-insulin-dependent diabetes. Metformin-induced shifts in cellular energy balance increase AMPK activity, enhancing insulin’s effects in the liver and skeletal muscle, thereby reducing hepatic glucose production and increasing peripheral glucose uptake and utilization. Metformin also increases cyclic adenosine monophosphate (cAMP) by regulating lipid metabolism, thus primarily enhancing insulin sensitivity, and counteracting the hyperglycemic effect of glucagon, thereby lowering blood sugar levels. Moreover, metformin increases glucagon like peptide 1 (GLP-1) secretion and induces the upregulation of GLP-1 receptors on the surface of insulin-secreting cells, enhancing glucose-dependent insulin release from the pancreas (95–97). AICAR, which activates AMPK, reduces the levels of ECM proteins in TGF-β1-treated pulmonary fibroblasts, alleviates TGF-β1-mediated resistance to apoptosis in lung fibroblasts, and enhances mitochondrial bioenergetics. Metformin accelerates the resolution of bleomycin-induced lung fibrosis, which is dependent on its activation of AMPK (98). Sato et al. also showed that the attenuation of bleomycin-induced PF in mouse model and the inhibition of myofibroblast differentiation by metformin is dependent on the suppression of enhanced Nox4 expression and Smad phosphorylation, and downregulation of TGF-β-induced NOX4 expression, respectively (62).

### Insulin

Insulin is the only hormone in the body that lowers blood glucose levels and is secreted by pancreatic β-cells. Its secretion is regulated by exogenous or endogenous substances such as glucose and glucagon. Insulin promotes the uptake of glucose by tissue cells and stimulates the synthesis of glycogen, fats, and proteins. Insulin decreased the mortality of rats in LPS-induced acute lung injury, and reduced LPS-induced inflammation in a PI3K/Akt-dependent manner without affecting blood glucose levels in mice (99, 100). Insulin also regulates epithelial sodium channel (ENaC)-mediated alveolar fluid clearance by inhibiting Nedd4–2 via the upregulation of the PI3K/Akt pathway in LPS-induced acute lung injury in rats (100). The PI3K/AKT pathway is an important regulator of PF. In IPF patients and bleomycin-induced PF in rats the PI3K/Akt pathway is significantly activated, and the specific PI3K/Akt inhibitor LY294002 reduces inflammation, reverses EMT, and significantly alleviates symptoms of PF. Currently, PI3K/AKT inhibitors are being evaluated clinically for IPF (101, 102). Therefore, insulin may also have crosstalk with PF.

### Insulin-like growth factors (IGFs)

IGFs are a class of broad-spectrum growth factors with 70% homology in chemical structure to insulin, and consist of insulin like growth factor 1 (IGF1) and insulin like growth factor 2 (IGF2). IGF1 stimulates glucose transport in muscle cells, inhibits hepatic glucose output, and lowers blood glucose by binding to insulin receptors; and it also plays a role in maintaining blood glucose levels during fasting (103, 104). IGF1 is upregulated by TGF-β in murine fibroblasts through the *Class-1 IGF1* promoter, and IGF1 and phosphorylated IGF1R (insulin like growth factor 1 receptor) are increased in bleomycin-induced murine fibrotic lung (105). Studies show that the expression levels of IGF1 and insulin like growth factor binding protein 3 (IGFBP3) are elevated in the bronchoalveolar lavage fluid of IPF patients, which is related to the early stages of the disease, and they can serve as clinical markers of the disease (106). IGF1 treatment increases the mRNA and protein levels of Glut4 and HK2 in human embryonic lung fibroblasts, as well as increases the expression of elastin and collagen IV (107). In a soft matrix environment, IGF1 stimulates the expression of genes such as *α-SMA*, *Col1a1*, and *Col3a1* in fibroblasts, thereby promoting their differentiation into myofibroblasts (108). Additionally, IGF1 enhances the survival and migration of lung fibrotic cells through the insulin receptor substrate 2/PI3K/Akt axis; treatment with the anti-IGF1 receptor monoclonal antibody (A12) to block the IGF pathway increases fibroblast apoptosis, and A12 reduces lung fibrosis in a bleomycin-induced animal model (109).

In lung fibroblasts associated with fibrosis in scleroderma/systemic sclerosis, the expression of IGF2 is higher than normal, and it promotes ECM production through pathways dependent on PI3K and Jun N-terminal kinase (110). Additionally, IGF2 regulates the expression of TIMP (metallopeptidase inhibitor 1), reduces MMP3 levels, thereby promoting a fibrotic environment intracellularly and extracellularly, and reducing extracellular matrix degradation in lung interstitial cells. IGF1R, insulin receptor (IR), and IGF1R/IR all mediate the differentiation of fibroblasts into myofibroblasts induced by IGF2 (111).

### Peroxisome proliferator-activated receptor gamma (PPARγ)

PPARs are a class of nuclear receptors involved in lipid metabolism, cell proliferation and differentiation, macrophage function, and immune regulation. PPARs have three subtypes: α, β/δ, and γ. PPARγ, is predominantly found in adipose tissue and the immune system, and promotes adipocyte differentiation and reduces inflammatory responses when activated (112, 113). PPARγ regulates the secretion of various cytokines and hormones, and the associated signaling pathways and biological processes, such as adiponectin, ghrelin and thyroid hormone (114–116).

PPARγ plays a critical role in carbohydrate metabolism. In intestinal epithelial cells, PPARγ regulates the expression of the *lactase gene*, improving symptoms of lactose intolerance in rats (117). It also enhances insulin sensitivity in peripheral tissues, thereby improving muscle glucose utilization and reducing hepatic glycogen output (118). Treatment with PPARγ agonist, PIO, has been found to improve insulin sensitivity in mice, ameliorate hyperglycemia and dyslipidemia, prevent excessive lipid accumulation in peripheral tissues, and to some extent, inhibit the production of pro-inflammatory cytokines (119). *In vitro* molecular docking analysis demonstrated that Rumex dentatus extract (RDE), which is rich in polyphenols, binds strongly to PPARγ, upregulating its expression, alleviating hyperglycemia, insulin resistance, carbohydrate metabolism disorders in type 2 diabetic rats, and inhibiting oxidative stress and inflammation *in vivo* (120).

PPARγ also plays an important regulatory role in glycolysis. It has been reported to drive activation-induced glycolysis in human T helper 9 cells (121), and mediate glycolysis in hepatocellular carcinoma cells through pyruvate kinase M1/2 (PKM2) (122), and act as a novel upstream regulator of the glycolytic enzyme ENO1 to promote glycolysis in bladder cancer cells (123). Additionally, antagonism of PPARγ signaling downregulates the expression of fructose-bisphosphatase 1, a negative regulator of glycolysis, without damaging mitochondrial function, thus expanding human hematopoietic stem and progenitor cells by enhancing glycolysis (124).

Research has shown that in the lungs of patients with IPF, the expression levels of PPARγ and its target genes are decreased (125). TGF-β can diminish the expression of PPARγ in lung myofibroblasts. Furthermore, the knockout of PPARγ leads to increased expression of α-SMA and collagen in these cells (126). PPARγ agonists, rosiglitazone and troglitazone have shown protective effects on bleomycin-induced pulmonary fibrosis in murine models (127, 128). Rosiglitazone and another PPARγ agonist, ciglitazone inhibited TGF-β1-induced EMT in alveolar epithelial cells, and their inhibition in collagen I and E-cadherin expression appeared to be PPARγ-dependent (129). Therefore, PPARγ agonists may be potential therapeutics for IPF with efficacy.

**Figure 1:**
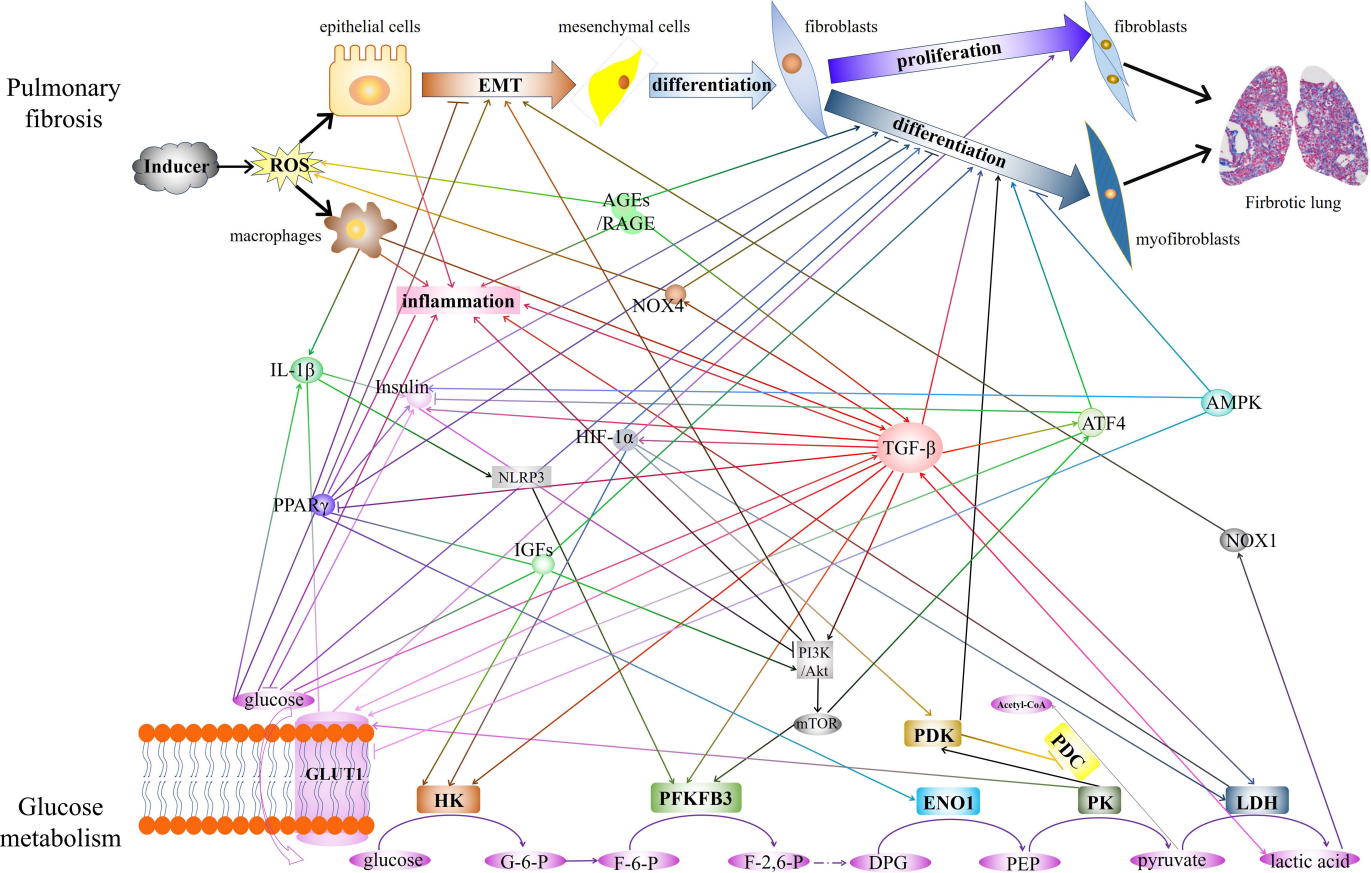
The diagram showed the close crosstalk between pulmonary fibrosis and glucose metabolism. The interactions of key enzymes, metabolite, and critical regulatory factors that play regulatory roles in glucose metabolism and pulmonary fibrosis are demonstrated. EMT, epithelial-mesenchymal transition. AGEs, Advanced glycation end products. RAGE, receptor for AGEs. IL-1β, interleukin-1 beta. PPARγ, peroxisome proliferator-activated receptor gamma. GLUT1, glucose transporter 1. NOX4, NADPH oxidase 4. HIF-1α, hypoxia-inducible factor 1 subunit alpha. NLRP3, NLR family pyrin domain containing 3. IGFs, Insulin-like growth factors. TGF-β, transforming growth factor-beta. ATF4, Activating transcription factor 4. AMPK, AMP-activated protein kinase. NOX1, NADPH oxidase 1. HK, hexokinase. PFKFB3, 6-phosphofructo-2-kinase/fructose-2,6-bisphosphatase 3. ENO1, enolase 1.PK, pyruvate kinase. LDH, lactate dehydrogenase. PDK, pyruvate dehydrogenase kinase. PDC, pyruvate dehydrogenase complex.

The diagram in **Figure 1** shows a summary of the close crosstalk between pulmonary fibrosis and glucose metabolism.

## Conclusions

Several risk factors contribute to the development of IPF, including diabetes, smoking, environmental exposure, genetic susceptibility, age, viral infections, and GERD. Among them, diabetes has been evidenced to be a causative factor. With the continuous advancement of science and technology, there is growing recognition of the close relationship between glucose metabolism and PF. Targeting key enzymes, glucose transporters, or metabolic products in glucose metabolic pathways, as well as altering related cytokines, can provide a basis for exploring new treatment methods for PF. In the near future, specific targeting of glucose metabolism may become an important means for the clinical diagnosis and treatment of PF.

## Author contributions

YW: Writing – original draft, Writing – review & editing. XW: Writing – original draft. CD: Writing – original draft. ZW: Writing – review & editing. JW: Writing – review & editing. NZ: Writing – review & editing. BW: Conceptualization, Writing – review & editing. KT: Conceptualization, Writing – review & editing. YF: Conceptualization, Writing – review & editing. PC: Conceptualization, Writing – original draft, Writing – review & editing.
